# Lessons learned from child health care nurses' experiences of teaching infant massage groups: A qualitative interview‐based study

**DOI:** 10.1002/nop2.1524

**Published:** 2022-12-07

**Authors:** Josefin A. Isaksson, Gerth Hedov, Pernilla Garmy

**Affiliations:** ^1^ Faculty of Health Sciences Kristianstad University Kristianstad Sweden; ^2^ Faculty of Medicine Lund University Lund Sweden

**Keywords:** child health care nurses, infant massage, interviews, qualitative study

## Abstract

**Aim:**

To describe child health care nurses' experiences of teaching infant massage in parent groups.

**Design:**

This was an exploratory‐descriptive qualitative study based on individual interviews.

**Method:**

Qualitative semi‐structured interviews were conducted with child health care nurses (*N* = 9) according to the COREQ guidelines and analyzed with qualitative content analysis.

**Results:**

Five categories were identified: (1) Infant massage can promote attachment between parents/guardians and their children; (2) Infant massage can have a calming impact; (3) Stress and lack of time can be challenging; (4) The composition of parent groups can be important and (5) The child health care nurse can observe parents'/guardians' relationships with their children. Child health care nurses are uniquely familiar with infant massage and the benefits it provides both parents/guardians and their infants. Specifically, infant massage has a calming effect that reduces stress and strengthens the relationship between infants and their parents/guardians.

**Patient or Public Contribution:**

Child health care nurses were interviewed.

## 1 INTRODUCTION

Infant massage involves strokes applied using different movements uniformly over the infant's body (Field, 2019). Its many documented benefits include promoting weight gain (Lestari et al., 2021; Lu et al., 2020), reducing jaundice (Abdellatif et al., 2020; Jazayeri et al., 2021), alleviating pain (Fitri et al., 2021; Gholami et al., 2021), increasing alertness (Hendy et al., 2022), and also preventing post‐partum depression in mothers (Pados & McGlothen‐Bell, 2019). Training in infant massage can be received in person in both group and individual settings (Midtsund et al., 2018) or online (Khuzaiyah et al., 2022). Parents/guardians can continue with their massage practice at home after the infant massage course (Menici et al., 2021). Most studies on infant massage focus on measuring its effects rather than the experiences of those involved in the process. These quantitative studies have been conducted around the world and found mainly positive effects on pain relief, weight gain and jaundice (Mrljak et al., 2022). Qualitative studies on child health care nurses' experience teaching infant massage in parent groups are currently lacking. Additional research is needed to further develop this activity in child health care.

## 2 BACKGROUND

Infant massage is a common worldwide practice (McClure, 2017). A recent review revealed that, most often, health care professionals teach infant massage to mothers in groups (Vicente & Pereira, 2021). The International Association of Infant Massage certifies instructors in infant massage after one‐week training with follow‐up (Weatherford, 2022).

Communication between parent/guardian and child is a crucial part of an infant massage as it occurs primarily through body language (Balakrishna et al., 2019). As a parent/guardian, observing and responding to the child's signals is essential. For example, if the infant becomes uncomfortable or upset, it is necessary to stop massaging. As a parent/guardian, it is imperative to observe these subtle signs from the infant throughout the massage, i.e., before the massage begins until after it ends (Field, 2019).

The overall goal of Swedish Child Health Care is to enable the best possible physical, mental and social health from newborn to school age. This is made possible by promoting the child's health and development while at the same time working to prevent children's ill health (Wettergren et al., 2016). Paying close attention to problems with children's health, development and home conditions enables implementing the appropriate measures early to ensure that the child receives the best possible care (Skoog, 2022). In Sweden, child health care nurses earn their master's degrees either as district nurses or paediatric nurses. They lead group‐based parental support classes (Lefevre, 2016), where infant massage sometimes occurs (Garmy, 2007, 2012). While the effects of infant massage are explored in the literature (Field, 2018; Mrljak et al., 2022; Pados & McGlothen‐Bell, 2019), there is a lack of qualitative research on the experiences of healthcare professionals, such as child health care nurses. Therefore, it is essential to expand our knowledge of the topic based on the unique perspectives of the nurses responsible for teaching infant massage to groups of parents/guardians. This invaluable insight can help inform other nurses and healthcare providers/administrators towards improving and expanding these training opportunities in child health care. The aim of the current study was to describe child health care nurses´ experiences of teaching infant massage in parent groups in child health care centres.

## 3 METHODS

### 3.1 Design

This was an exploratory‐descriptive qualitative study based on individual interviews. The study was conducted according to the COREQ guidelines (Tong et al., 2007). An inductive methodological approach was used to examine the informants' lived experiences. The study was approved by the Swedish Ethical Review Authority.

### 3.2 Procedure

A purposeful sampling technique was used (Shaheen & Pradhan, 2019). Managers of child health care settings (*N* = 87) were contacted by e‐mail. After obtaining their consent (*N* = 10), informative letters and consent forms were sent to child health care nurses (*N* = 24). This yielded nine informed, consenting participants, see Table 1, which led to the interviews that generated the data analyzed in this study.

**TABLE 1:** Recruitment process.

	Contacts	Giving consent	Declining	No response	Number of reminders sent
Managers (*n*)	87	10	8	68	1
Child health care nurses (*n*)	24	9	4	11	1–2

### 3.3 Sample and context

Inclusion criteria were nurses with at least 1 year of experience in teaching infant massage to parent groups in Child Health Care settings in southern Sweden. The informants were nurses for 6–36 years (Table 2). They had also worked for different lengths of time at their current workplaces, ranging from 1 to 23 years. The informants all received training in infant massage certified by the International Association of Infant Massage (IAIM), and all classes were taught according to the IAIM method. The infant massage classes for parents were organized by the child health care centre, delivered by the child health care nurse, and were free of cost. Classes held more seldom, and in smaller groups during the pandemic. Classes were not offered online. Typically, groups consisted of 3–10 infant – parent/guardian dyads. The infants were about 2–6 month, and the classes were held about once a week for 1–2 h a time, for 3–8 weeks.

**TABLE 2:** Participant description (*n* = 9).

Interview	Masters education for the child health care nurses	Numbers of years working in child health care	Numbers of year as an instructor of infant massage	Organization of the child health care setting
1	District nurse	10	7	Private
2	Paediatric nurse	8	3	Private
3	District nurse	15	12	Private
4	District nurse	13	8	Private
5	District nurse	12	12	Public
6	Paediatric nurse	9	3	Private
7	District nurse/paediatric nurse	36	36	Private
8	District nurse	23	13	Public
9	District nurse	6	3	Private

### 3.4 Data collection

The first author conducted nine qualitative semi‐structured individual interviews from May to October 2021. The benefit of semi‐structural interviews is that the same questions are asked in all interviews, while the semi‐structural format enables a comfortable dialogue (Brinkmann & Kvale, 2018). The informants were informed orally and in writing that the interviewer (the first author) was a female registered nurse studying to achieve her master's degree in district nursing. Because of the COVID‐19 pandemic, the interviews were conducted with the digital tool Teams. The interview guide (Appendix 1) consisted of five background questions and three open‐ended general questions to provide an opportunity for more in‐depth, descriptive answers where the respondents were asked to relay their experience of teaching infant massage to groups of parents. An example of questions in the guide was: “What can you see for the pros and cons of teaching infant massage in groups?” and follow‐up questions such as “Can you tell me more” and “Can you give examples.” Written informed consent from the participating nurses was obtained. Two pilot interviews were conducted to ensure that the interview questions were relevant; however, since the pilot interviews were rich and the interview guide functioned well, no adjustments were made. The median duration of the interviews was 40 min. The interviews were recorded with a dictaphone. The recordings were then transcribed verbatim into text by the first author. The transcribed text consisted of 25,000 words.

### 3.5 Analysis

Qualitative content analysis was used (Lindgren et al., 2020). The analysis process was performed on a manifest and latent level. The transcribed texts were read by the authors several times. Initially, sentences from the interviews that responded to the aim were extracted and needled up on a large screen to provide an overview of the work. Thereafter, the extracts were reduced to even smaller units of meaning where the words of meaning remained, which is called condensation. Subsequently, text with similar meanings were grouped into subcategories and categories (Lindgren et al., 2020). This process was repeated several times until the categories felt right. This analysis was discussed among the authors until a consensus was obtained. An example of the analysis is provided in Table 3.

**TABLE 3:** Example of the analysis process.

Extracts from the interview	Condensed text	Subcategories	Categories
Oh! Yes, I mostly see the advantages, that is, it's a cozy moment with the child, sitting with the child, creating contact with the child, that's what I see there anyway	Cozy moment with the child, sit with the child, create contact with the child	It enables increased proximity to one's child	Infant massage can promote attachment between parents/guardians and their children
Well, then there is the fact that it is both relaxing for both the child and the parent. Because you get relaxed when you massage	Relaxation for the child and the parent. You become relaxed when you massage	Infant massage often has a relaxing effect	The infant massage can have a calming impact
Eh, I tell the parents that after infant massage some babies get a little more tired and sleep a lot that day	More tired and sleep a lot that day	Infant massage can have a positive tiring effect on the infant	The infant massage can have a calming impact

## 4 RESULTS

Five categories were identified from the results (Table 4). The child health care nurses' experiences with teaching infant massage in parent groups were broad and extensive. While their experiences were different in many respects, most of them shared common or similar features. Experiences, such as the impact of infant massage on attachment, its calming effect, and its sensitivity to stress and lack of time. All nurses described how situations and individuals could be important for the infant massage groups and the parents'/guardians' ability to take in and learn the infant massage.

**TABLE 4:** Categories and subcategories identified in the results.

Categories	Subcategories
Infant massage can promote attachment between parents/guardians and their children	It enables increased proximity to one´s child
	It gives the parent a deeper understanding of their infant
The infant massage can have a calming impact	Infant massage often has a relaxing effect.
	Infant massage can have a positive tiring effect on the infant
Stress and lack of time can be challenging	Lack of time can adversely affect the performance of the infant massage
	Stressed parent/guardian negatively affect their infant
The composition of parent groups can be important	The group creates a sense of community
	The willingness to participate varies
	There may be some inequality in infant massage groups
	The arrangement of the infant massage may vary
The child health care nurse can observe parents'/guardians' relationships with their children	Enables the child health care nurse to gain insight into the emotional relationship between parent/guardian and infant
	Provides an opportunity to pay attention to how the parent/guardian touching the infant
	It enables the child health care nurse to get to know the infant's parents/guardians better

### 4.1 Infant massage can promote attachment between parents/guardians and their children

Most nurses talked about their experiences with attachment and how they believed that infant massage could promote and help parents/guardians attach to their infants. One of them described her experience as follows:

Not all parents have a great connection to their children, but this promotes the connection because you have to look at your child when you do infant massage and it can be a very positive experience and a very nice connection. (nurse 6)

Some of the nurses described the importance of parents/guardians touching their infant and the value of the closeness derived from skin‐to‐skin contact. One nurse talked about how important it was to be content with touching:

Just put your hands and like not massage but just take the touch and then the child usually calms down so that you see how positive it is for both because you see the parents sitting and talking to their child. (nurse 3)

Another nurse relayed how parents/guardians touched their infant entirely differently during the infant massage than they usually did at home. In everyday life, the nappy is changed, and the infant is bathed, yet during the massage, they touched the whole infant, and the nurse thought this was good.

Some nurses talked about first‐time motherhood and how difficult it could be to know whether you were doing the right thing or if your baby even liked you. One nurse narrated an event that she will always carry with her:

I had a mother once who had some problems with her first child, she was afraid of making mistakes, but she started massaging. She was very sad, got help from a psychologist and such, but she started massaging her child and then we met after a week and then something had happened. It kind of happened in connection between the two, the child liked it, oh well then the mother felt that the child might like me, I'm doing right. (nurse 5)

The nurses described the importance of seeing their infant, getting to know the infant's signals and being able to read them. In the infant massage groups, parents/guardians had to practice this. If the infant became bothered or sad, they had to pause the massage and listen to the infant. One nurse described how she experienced parents/guardians learning to be aware of their infant, getting to know their infant's signals, and how respecting the infant is part of the massage process. Another nurse talked about the importance of paying attention to the infant using non‐verbal dialogue, observing how they react to different touches, and respecting the infant's feelings, which she described as follows:

Every time you are going to massage you ask for permission, look the child in the eyes, eh while you ask for permission, for example, ‘May I massage your legs?’ so you have oil on your hands and you do this with the massage oil [rub your palms against each other] and showing the hands to the child, the child will recognize it. Every time it looks the same, and if you notice that the child turns his head away or is whining or crying, you must wait. (nurse 9)

The nurses described the experience of connection as contact and closeness between parents/guardians and their infants. One explained:

Yes, and so infant massage is a way for fathers to get close, although they are not breastfeeding. (nurse 1)

### 4.2 Infant massage can have a calming impact

The nurses expressed that infant massage in parent groups often had a calming effect on the parents/guardians and their infants. One of them described how the infant massage is a cozy moment with calming effects that make one relax.

One of the nurses explained that the infant massage had a calming effect even on her:

I can feel that when you sit and massage the instructors' doll [in the infant massage group], I can feel how you relax yourself. And how that moment becomes as rewarding. (nurse 6)

One nurse explained how she experienced being able to return to the infant massage groups' calm atmosphere when parents/guardians are stressed and worried about their infants. She recalled that reminding parents/guardians of how they felt during the infant massage allowed them to find that calm again:

When we had the course in infant massage, it could be an advantage if you could be calm yourself and embrace that feeling of calm and that you have set aside time to do this, and then it is just us here and now. (nurse 9)

Another nurse reflected on how she benefited from the infant massage that one of the thoughts with this massage is that the parents/guardians would, after the group session, continue to massage their infant at home. This technique could be utilized by parents/guardians when their infant requires yet resists rest by helping the infant fall asleep and ensuring the infant sleeps peacefully and soundly for a longer duration.

Many nurses described the group session as a relaxing moment for both the infant and their parent/guardian. When reflecting on the benefit of infant massage, oxytocin was often mentioned, particularly how the experience was soothing and calming due to the release of oxytocin. One nurse recounted explaining to the parents/guardians that the infant could be calmed by the massage:

I tell the parents when they have done the baby massage that their children may be calmer, a little tired and sleep a lot that day. (nurse 4)

### 4.3 Stress and lack of time can be challenging

Some of the nurses mentioned that stressed parents/guardians could affect their infants negatively, so an otherwise soothing infant massage instead became something that made the infants sad, which could cause even more stress and anxiety in the parents/guardians. The nurses experienced that parents/guardians today are stressed; they have so many routines around the babies and the family. Nurses observed a lack of time in today's families. They found that people had too much to do and put a lot of focus and time into social media and other things in life. Parents/guardians seemed to have a hard time finding a quiet moment for infant massage among all the routines, shopping, breastfeeding and sleep periods. The nurses also talked about how the parent's/guardian's stress affected their infant negatively. An example was a mother who, the first time she came to the infant massage class, had been relaxed, and the infant enjoyed the massage. However, the next time she came, things had not gone so well when the mother immediately told the nurse that she did not really have time to be there but that she had come anyways, which the nurse described as follows:

The child felt it because from the time she undressed the child until she left, the child screamed all the time, so it was as clear as her stress there as if it had spread to the child. (nurse 8)

One nurse said that it was disadvantageous for the infant massage group if parents/guardians were stressed. She found that when parents/guardians were on their way somewhere and were not present in the moment, the massage experience suffered. Such instances required explaining the importance of being engaged in the moment, and after the massage, then they could move on. She experienced that this reminder would make them relax. Another nurse talked about experiences in which parents/guardians came to the infant massage groups stressed and sometimes late. She concluded:

It [infant massage] should be relaxing and of course, the parents stress in and out and then you should get rid of clothes. I feel that the children became quite worried about it and I often feel that the parents are quite stressed. (nurse 2)

One of the nurses mentioned the length of the infant massage sessions as a reason why parents/guardians chose not to prioritize the massage when time ran out. She also talked about how parents/guardians did not massage their babies at home as intended, the reason for this being a lack of time and that the family was busy with everyday life, which they considered a higher priority than the infant massage.

### 4.4 The composition of parent groups can be important

All nurses shared experiences about how the composition of participants in the infant massage groups was important. Several of the nurses described their experiences of how parents'/guardians' intentions and their opportunities for participation varied. They talked about it being more common for the mother to be present at the infant massage as it was usually the mother who was at home with the infant at that age. One nurse described it like this:

The most common are mothers, they are on parental leave as we have infant massage in parent groups. It is a prerequisite that the children are small when we start this. (nurse 9)

The nurses expressed that both parents/guardians were invited to the infant massage groups but that it was usually the mother who came. Sometimes the father had also come, but then, as a rule, the mother had also been involved, and the father had seldom come by himself. One nurse said that the few times when fathers came themselves when the mother had started working again. Another nurse talked about a specific situation where both parents were involved in the infant massage. In that case, they had shared being at home with the baby. She explained it as follows:

Have had a father who has been in a group regularly, ehm but there they had shared being at home, ehm and the mother did not breastfeed, and it is perhaps breastfeeding that makes the women who are usually at home. (nurse 4)

She talked further about a situation where both the mother and the father participated in the infant massage class. As they had only one infant, the father practiced the massage on a doll while he sat close to his infant and her mother. This dad did not join the class thereafter for reasons the nurse did not know for sure, but she thought it could be because he had not liked massaging a doll.

Some of the nurses mentioned a recent change they observed in the demographics of the group. The fathers have been more at home after the babies are born, and they are taking paternity leave in a different way than before, which led to the fathers being able to be more involved in parent groups than they have been in the past. The nurses thought this was good because it made it allowed them to get to know the fathers through conversations with them. But it is still more common for the mother to come to this type of parent group.

One nurse talked about her experiences of working with motivation. She tried to make the parents/guardians understand the importance of them *both* performing the infant massage and that the father could perform it at home if it were he who worked and was, therefore, unable to join any of the group sessions.

One of the nurses talked about her experience about the participation of same‐sex couples, which could differ from heterosexual couples where she has never met a father in the infant massage groups. She described it like this:

In same‐sex couples, it is more common for the non‐birth mother to take part in the baby massage and is the one who massages, and sometimes both come, and sometimes the one who gave birth has come and sometimes the other way around. (nurse 2)

Some of the nurses highlighted that it was not apparent that all parents/guardians or their infants wanted to participate in the group activities. One nurse talked about how the reluctance to participate in an infant massage group involving instances when the babies would become sad and start crying, thereby disturbing the other babies, and leading them to not want to be part of the parent group activity. One nurse recounted how some parents/guardians do not feel comfortable in a group context. Specifically referring to those who had diagnoses of some kind; she said:

I've had someone who might have a diagnosis or something who thinks it's hard in a group, and then I've had to show in, so individually then, eh just because they feel they do not want to. (nurse 3)

Some of the nurses shared their experiences of the community fostered in the infant massage groups, and how this type of parent group can bring parents/guardians together to learn from each other. As one of the nurses described:

This happens in a group where it is that way because we are sitting on the floor, I lay out mattresses, we light candles, listen to music. You sit together, but not now during the COVID‐19 pandemic [laughs], then it should be two meters apart, but before that you sat very tight, the children undress, eh so that eh it's cozy. (nurse 5)

Some of the nurses recounted experiences of the community in the infant massage groups and how this type of parent group brought them closer together. Some of the nurses talked about how parents/guardians would take part in each other's questions, that one could ask a question that the others had thought about but did not dare to ask. This created a sense of community; they relaxed upon seeing that no questions were stupid. Being able to ask questions without hesitation allowed them to focus on the infant massage instead.

Other nurses said that parents/guardians got to know each other, looked at each other, learned from each other, and got new ideas. As one nurse explained:

Maybe some things are difficult but then you see that maybe the person sitting next to you is doing something different and think that maybe I can test, you get some ideas eh while you get to know some new people. (nurse 3)

One nurse talked about parents/guardians getting to know each other, making contacts and exchanging phone numbers to keep in touch after the infant massage sessions were over. Specifically, she recounted:

They get to know each other, that the mothers or fathers can make contact with other parents. As they can get support and advice from and get to know and continue to hang out with since. (nurse 1)

Some of the nurses talked about the arrangement around the infant massage groups. One nurse said that she used to hold five sessions to which about 10 people were invited. Sometimes only a few would come, and then they drove with the few who came. Another said that she held these courses four times per year, which involved two or three sessions with each parent group.

Another nurse mentioned a lack of space, which required her to adapt the number of participants in these infant massage groups to the room. She described her experience as follows:

It can be difficult with space and so when you become too many, but we have tried, we have made groups that are manageable, everyone should have a chance to ask questions and so… You should have time to tell so we have always invited a maximum of eight, and then maybe not all eight will come, so then it tends to be quite a suitable group, so it is quite appropriate to be like those six or seven participants. (nurse 9)

One nurse talked about her problems getting foreign parents/guardians to attend the infant massage groups. While they may have said yes, they then did not actually attend. She described these concerns as follows:

It is very difficult to reach foreign born mothers, do not think they really understand the thing, think that they probably also massage naturally, from different cultures so it is not something you kind of go away to do but you do it at home. (nurse 5)

### 4.5 The child health care nurse can observe parents'/guardians' relationships with their children

The nurses reported that they would sit during the infant massage groups and observe how parents/guardians engaged and interacted with their babies or answered questions that parents/guardians had about how to take care of their infant and what they liked. One nurse described observing how the parents lifted their baby or whether they had any bruises. She describes this aspect as follows:

You always have a small eye on everyone, you do not sit and watch, but you cast an eye how does the mother touch the baby. A bit like that! How to take care of your child. (nurse 7)

One nurse explained that while the group was massaging, she gained insight into families by reflecting on the questions that parents/guardians asked when they were unsure how to handle their infant. She saw this as an opportunity to give parents/guardians knowledge about their infant. Another nurse relayed something similar; she got to know the parents/guardians by asking them more extended questions and also by allowing them to ask her the questions they wanted.

Another nurse relayed how she used infant massage group opportunities to observe how the parents/guardians interacted with their infants. In these moments, she was able to gain insight into how parents/guardians and their infants felt together. She saw this as an advantageous opportunity to observe and get to know the families in this way. Similar experiences possessed another nurse who talked about sitting and observing the infant massage, answering parents'/guardians' questions. She seemed to get to know the families better when such observations and conversations took place under more relaxed conditions. She described it as:

In a group, you can sit and observe them, it becomes a different matter, and you get other questions, you can get to know the parents in a completely different way. Ah you get to know them well actually. (nurse 3)

Another nurse recounted her experiences with attachment problems and how important it was for her as a health care professional to be observant and sensitive to this concern during the infant massage group sessions. In these situations, she had to consider following up with the mother on an individual basis later, either in person or on the telephone, and not in front of the whole group right there and then.

## 5 DISCUSSION

The study describes the nurses' experience of teaching infant massage in parent groups. Several important findings came to light in this interview‐based study, and three of these key findings will be discussed here. The first finding was that, according to the nurses, infant massage in the parent group promotes attachment between parents/guardians and their children. The other central finding was that infant massage could have a calming effect on all who participated. The third finding indicated that the nurses experienced the composition of the parent groups as being important.

The nurses in this study described how infant massage in parent groups promoted the connection between parents/guardians and their infants. They believed that the massage gave the parents/guardians an opportunity to get closer to their infant; the massage provided a cozy time together where the babies and parents/guardians got to know each other, what they liked, what signals they gave, and what these meant. The bond between them became stronger. In an interview‐based study by Midtsund et al. (2018), the mothers described how their contact and connection to the child improved during the massage when they felt that they received a positive response from their infant. Moreover, from their analysis of parents/guardians' experiences, Chan et al. (2018) concluded that the infant massage enhanced attachment when the mother learned to communicate more adequately with her infant (Chan et al., 2018). A study in which they interviewed different focus groups revealed how nurses provide mothers with knowledge about their children's signals and states of mind so that the mothers could more easily understand and connect to their infants, so they could read their signals and use their body language to determine whether they liked the touch or not (Underdown et al., 2013). Midtsund et al. (2018) described how anxiety about colic and difficulties with feeding can negatively affect maternal attachment, while infant massage can help mothers rekindle a sense of attachment to their infants (Midtsund et al., 2018).

The nurses in this study described how infant massage had a calming effect on those who participated in the sessions. This health‐promoting impact is not limited to parents/guardians and their infants but also extends to the nurses who noticed their stress levels decreasing and becoming calmer and more relaxed when they taught massage in these groups. The nurses in this study also mentioned that infant massage tended to make the child more tired, of particular benefit for children with difficulty winding down in the evening. In a study by Chan et al. (2018), most mothers shared similar accounts of their children becoming significantly more relaxed from the infant massage and getting better sleep as the massage made them more tired. Children with a history of waking up frequently during the night woke up fewer times after they received a massage (Chan et al., 2018). The data collected in this study indicate that the closeness between parents/guardians and their infants fostered during an infant massage could promote mental health through its stress‐reducing, calming effects.

Participation in the parent groups was mentioned by all the nurses in this study in one way or another. Some spoke about how the mothers got to know other mothers during these group meetings, thereby establishing contacts that persisted long after the infant massage sessions. Underdown et al. (2013) revealed that infant massage groups gave mothers a chance to expand their social network. Since some mothers were isolated in their homes, these classes served as opportunities for them to get out and meet people. The mothers talked to and learned from each other (Underdown et al., 2013). This was echoed by the nurses in this study when they recounted how parents/guardians asked questions that the others took part in and watched each other and got ideas on how to touch their infant.

### 5.1 Strengths and limitations

We will discuss the strengths and limitations of the study based on the four criteria of rigour: credibility, transferability, dependability and confirmability (Graneheim & Lundman, 2004; Lincoln & Guba, 1985; Polit & Beck, 2021). Credibility is strengthened by the individual interviews where the participants can express their experience of teaching infant massage in‐depth. Due to the COVID‐19 restrictions, the interviews were conducted digitally, which might be a limitation. However, the interviewed child health care nurses were all familiar with the digital tools and did not have any negative feedback concerning the format. The transferability of the findings is limited by the respondents all being women from health care centres in Sweden. However, the method of teaching infant massage in parent groups is the same globally, and, therefore, the results should be applicable to other countries and regions. Dependability refers to the stability of the study. The semi‐structured interview design ensured that the same questions were asked in all interviews. Also, one interviewer (the first author) conducted all interviews, which strengthens the dependability of the study. Confirmability deals with the objectivity of the study. We used an inductive approach, and the interview guide included questions about both possible negative and positive experiences of infant massage, which strengthened the study. The analysis was conducted by all three authors, which ensures a higher level of objectivity than had only one author performed the analysis.

### 5.2 Implications for future research

The study was conducted among female child health care nurses in southern Sweden. Therefore, studies in other contexts might be needed. Given that this study concerned health care professionals' experiences, further studies that focus on guardians'/parents' views are warranted, and studies observing the infant massage group classes led by child health care nurses.

## 6 CONCLUSIONS

Through their professional experiences, child health care nurses have found that infant massage in a parent group setting confers several benefits to both parents/guardians and infants. It has a calming effect that reduces stress, and it strengthens the relationship between infants and parents/guardians. However, these parent groups can vary greatly in terms of participation. In general, there were more mothers than fathers who attended, foreign‐born families were more difficult to engage, and not everyone was comfortable in this type of social context.

## 7 RELEVANCE TO CLINICAL PRACTICE

Based directly on their first‐hand experiences, child health care nurses reported that infant massage in parent groups had a positive impact on families' well‐being and is an integral part of child health care. Child health care nurses found it beneficial to teach infant massage in parental groups.

## AUTHOR CONTRIBUTIONS

Study design: PG, GH, JAI; data collection: JAI; analysis: JAI, PG and GH; writing first draft: JAI; reviewing and agreeing to the final version of the manuscript: PG, GH, JAI.

## FUNDING INFORMATION

The study has been funded by the Gyllenstiernska Krapperup Foundation.

## CONFLICT OF INTEREST

None.

## ETHICAL APPROVAL

The study was approved by the Ethical Review Board at the Faculty of Health Sciences, Kristianstad University (U2021‐2.1.12‐831) and the Swedish Ethical Review Authority (2022‐00610‐01).

## Supporting information


Appendix S1

## Data Availability

The data that support the findings of this study are available on request from the corresponding author. The data are not publicly available due to privacy or ethical restrictions.

## APPENDIX 1

### INTERVIEW GUIDE

**Table:**

Background questions
How long have you been working as a child health care nurse? How long have you worked at this childcare center? What does the child health care organization look like where you work? What educations/courses have you attended to teach infant massage in a parenting group? How much experience do you have teaching infant massage in groups?
Open‐ended questions
What can you see for pros and cons of teaching infant massage in groups? What impact can you see infant massage having on the child and parent/guardian? How does infant massage help you in your work with the child and parents/guardians? *Follow‐up questions* Can you tell me more Can you give examples
